# The Dietary Inflammatory Index and Sarcopenia in Older Adults in Four Chinese Provinces: A Cross-Sectional Study

**DOI:** 10.3390/nu17030478

**Published:** 2025-01-28

**Authors:** Rongchang Pu, Qingqing Man, Shuang Song, Shanshan Jia, Zhen Liu, Xiaona Zhang, Jian Zhang, Pengkun Song

**Affiliations:** 1 Department of Elderly and Clinical Nutrition, National Institute for Nutrition and Health, Chinese Center for Diseases Control and Prevention, 27 Nanwei Road, Xicheng District, Beijing 100050, China; prc052100@163.com (R.P.); manqq@ninh.chinacdc.cn (Q.M.); songshuang@ninh.chinacdc.cn (S.S.); jiass@ninh.chinacdc.cn (S.J.); liuzhen@ninh.chinacdc.cn (Z.L.); zhangxn@ninh.chinacdc.cn (X.Z.); zhangjian@ninh.chinacdc.cn (J.Z.); 2 Key Laboratory of Public Nutrition and Health, National Health Commission of the People’s Republic of China, Beijing 100050, China

**Keywords:** sarcopenia, dietary inflammatory index, cross-sectional study

## Abstract

**Background**: Sarcopenia associated with aging severely affects the quality of life of the elderly; diets have been shown to elicit an inflammatory response in the body, and diets that promote inflammation may lead to damage to muscles. The Dietary Inflammatory Index (DII) has been developed to quantify the inflammatory potential of individual diets. Therefore, this study aimed to investigate the association between the DII, sarcopenia and its components in elderly Chinese people. **Methods**: In this study, data were obtained from the China Nutrition Improvement Strategies and Applications for the Elderly Research Programme. An assessment of sarcopenia was carried out according to the Asian Working Group on Sarcopenia’s (AWGS2019) criteria. The DII was calculated using food intake data obtained using the FFQ method, and then the quartile method was used to categorize the subjects into four groups. Multifactor logistic regression was conducted to examine the associations between DII, sarcopenia and its components. **Results**: This study included 993 subjects over the age of 65, and the prevalence of sarcopenia was 20.2%. The mean DII score of the study population was 0.99 ± 0.1. After adjusting for the confounders age, gender, marital status, and educational level, the risk of sarcopenia was 1.66 times higher in group Q4 than in group Q1 (*p-trend* < 0.05). However, this relationship is not statistically significant when other more confounding factors are added. Nevertheless, when further analyzing the relationship between DII and sarcopenia components, it was found that after adjusting the model, a higher DII was associated with a risk of muscle strength loss (OR = 1.65, *p-trend* < 0.05). **Conclusions**: Higher DII scores increase the risk of muscle strength loss in older adults. By guiding older adults to adopt a more anti-inflammatory diet, muscle health can be improved in terms of increased muscle strength. Further cohort or interventional studies are necessary to validate our findings.

## 1. Introduction

Sarcopenia, defined by a decrease in skeletal muscle mass and muscle strength or physical function in older adults, significantly elevates the risk of falls, fractures, disability and mortality [1]. The global prevalence of sarcopenia in the elderly remains high and the development of sarcopenia is influenced by a variety of factors, including environmental conditions, genetic, nutritional status, physical activity, systemic chronic inflammation and oxidative stress [2]. Current research has not yet found a cure for sarcopenia, but a number of interventions have been explored, primarily to effectively prevent sarcopenia from occurring and prevent disease progression by preserving muscle mass and function [3].

Among the multiple factors influencing sarcopenia, the level of inflammation in the body, especially chronic low-grade inflammation, is thought to play an important role [4]. With age, cellular senescence and the accumulation of reactive oxygen species can result in chronic inflammation, usually characterized by elevated levels of blood inflammatory markers in cells and tissues [5]. Current research has identified inflammation as a risk factor for various diseases, including diabetes [6], dementia [7] and cardiovascular disease [8]. Its impact on muscle health is mainly reflected in the fact that the reduction in skeletal muscle mass in inflammatory states is associated with an increase in pro-inflammatory cytokines such as Tumor Necrosis Factor-α, Interleukin-6 and CRP, which amplify proteolytic metabolism and inhibit protein synthesis in skeletal muscle, thus leading to muscle wasting disorders [9]. Conversely, anti-inflammatory cytokines can reduce muscle atrophy by antagonizing the effects of pro-inflammatory cytokines [4]. Therefore, measures to regulate the level of inflammation are important in both the prevention and treatment of sarcopenia.

The level of inflammation in the body can be influenced by various factors, and dietary factors receiving increasing attention as modifiable lifestyle factors related to inflammation [10]. Available findings suggest that the intake of single nutrients such as Ω-3 polyunsaturated fatty acids, dietary fiber and vitamin E [11] is associated with reduced levels of inflammation. Conversely, the intake of nutrients such as total fat, carbohydrates and saturated fatty acids can increase the degree of inflammation [12]. However, complex interactions between nutrients in the dietary structure may alter the true effect of specific nutrients on inflammatory responses or health outcomes [13]. Consequently, recent years have seen numerous studies employing both a priori and a posteriori methods to investigate the relationship between dietary patterns and inflammation. And more representative results suggest that a Mediterranean diet rich in vegetables, fruits, olive oil and unsaturated fatty acids is thought to reduce the levels of inflammation and is associated with a reduced risk of developing a number of chronic diseases [14]. In contrast, the Western dietary pattern, which is characterized by a high intake of red meat, saturated fatty acids and processed foods, is associated with increased levels of inflammation [15].

As the role of diet in inflammation and inflammation in health becomes better understood [16], a reliable indicator of the inflammatory potential of the diet is increasingly important for scientific research and disease prevention. As a result, the Dietary Inflammatory Index (DII), which is used to assess the overall inflammatory potential of an individual’s diet, has been developed and utilized [17,18]. The DII, which has been refined and revised since its introduction, is now being used in a large number of clinical studies, and the results of the available studies suggest that the DII is directly linked to inflammatory biomarkers [19]. In addition, the DII has been associated with many health outcomes, particularly chronic diseases associated with inflammation in the body like cardiovascular disease [20], dyslipidemia [21] and metabolic syndrome [22]. Regarding the relationship between DII and sarcopenia, despite strong evidence confirming the connection between inflammation and diet as well as sarcopenia, only a limited number of studies have investigated the link between dietary inflammatory potential, sarcopenia and its components, and there is some variation in the findings [23]. The results of existing relevant meta-analyses suggest that the DII may be linked to muscle mass, muscle strength and the development of sarcopenia [24,25]. However, most of the existing studies on this topic has been conducted mainly in Europe and the United States, and most of the current studies based on the Chinese population are limited to small sample surveys in a certain region [26,27]. Dietary habits also vary greatly between different countries and regions, so more epidemiological evidence for populations in different regions is needed. Therefore, the aim of this study was to explore the link between dietary inflammatory potential and muscle health in four regionally representative community-dwelling elderly populations in China. Through this association we can identify the role that dietary inflammatory effects play in muscle decay in older adults, thus providing theoretical support for the dietary management of sarcopenia from an anti-inflammatory perspective.

## 2. Methods

### 2.1. Participants

This study comes from the China Nutrition Improvement Strategies and Applications for the Elderly Research Programme, a cross-sectional study conducted in 2018 and 2020. This project selects survey sites based on factors such as the major dietary habits of the geographic area, overall food intake characteristics and the capacity of grass-roots public health services, as well as the results of analyses of data from the monitoring of the nutritional and health status of the Chinese population. Four areas with typical dietary characteristics, Guangzhou City in Guangdong Province, Bayanaoer City in Inner Mongolia Autonomous Region, Taicang City in Jiangsu Province and Wuhan City in Hubei Province, were ultimately selected as typical survey sites. In accordance with the principle of random sampling, two to three communities from each survey site were selected to recruit elderly study participants. Individuals were excluded if they were (i) under 65 years of age; (ii) not permanent residents of the area; (iii) suffering from major physical or mental illness; (iv) had communication barriers that prevented them from completing the questionnaire; or (v) were limited in terms of the daily activities they could complete, disabled or unable to complete basic measures of physical fitness and body composition. A total of 993 elderly subjects were included in this study, and all participants signed an informed consent form before the survey. This study was approved by the Ethics Committee of National Institute for Nutrition and Health, Chinese Center for Disease Control and Prevention, with the ethics number 2018-014.

### 2.2. Questionnaire and Assessment of DII

The survey of the elderly consisted of a survey of basic personal information, living conditions, physical activity and a personal dietary survey. Personal information, disease history, lifestyle behaviors and other information were obtained by trained investigators during questionnaire interviews. The dietary survey used the Food Frequency Questionnaire (FFQ) to investigate the food consumption of the elderly in the past year, and the consumption of oil and condiments in the household of the elderly was also investigated. The 64 food items involved in the FFQ dietary survey were combined into 21 food groups based on the food composition table, and the consumption of each food group in the study population was analyzed. The dietary intake of the surveyed population was obtained through FFQ, and 100 g of each food was converted into its corresponding nutrient content according to the 6th edition of the “China Food Composition Table (Standard Edition)” [28]. These include calories, protein, fat, carbohydrates, dietary fiber, cholesterol, vitamin A, beta-carotene, thiamine, riboflavin, vitamin B6, vitamin B12, niacin, vitamin C, vitamin E, vitamin D, magnesium, iron, zinc, selenium, folic acid, alcohol, saturated fatty acids, monounsaturated fatty acids, polyunsaturated fatty acids, *n*-3 fatty acids and *n*-6 fatty acids, leading to a total of 27 nutrients. The above 27 nutrients were included in the calculation of the DII scores. Although the calculation of all 45 food nutrients in the DII was not addressed in this study, there are studies that confirm that the DII is still valid even if the number of nutrients used to calculate the DII is less than 30. The computation of the DII was performed in a stepwise manner following the instructions by Shivappa [18].

### 2.3. Anthropometric Measures

Anthropometric measurements were carried out by trained investigators. Height and weight were measured once using a stadiometer and an electronic weighing scale, and the data obtained were used to calculate the subject’s body mass index (BMI). The BMI was calculated using the formula weight (kg)/height (m)^2^. Waist circumference was measured twice using a soft leather ruler. The mean data of the two measurements were used for analysis.

### 2.4. Measurement and Diagnosis of Indicators Related to Sarcopenia

The diagnostic criteria for sarcopenia recommended by the Asian Working Group for Sarcopenia in 2019 (AWGS2019) [29] were utilized to determine whether the subjects had sarcopenia. Muscle strength was measured by grip strength and physical performance was represented by gait speed. Subjects with both low muscle mass and low muscle strength or low physical performance could be diagnosed as having sarcopenia.

#### 2.4.1. Muscle Mass

Body composition was measured by the bioresistive antibody method Inbody-770 (Biospace, Seoul, Korea). The skeletal muscle mass index (SMI) was calculated as appendicular skeletal muscle mass (ASM)/height (m)^2^. A low skeletal muscle mass, as defined by the diagnostic criteria for sarcopenia components recommended by AWGS 2019, is indicated in males with a skeletal muscle index (SMI) of less than 7.0 kg/m^2^ and in females with an SMI of less than 5.7 kg/m^2^.

#### 2.4.2. Muscle Strength

Grip strength was measured by an electronic grip strength meter (CAMRY EH101, Xiangshan, Zhongshan, China). Handgrip strength was measured twice for each hand in the standing position, and the greater recorded value was considered the maximal grip strength. Low grip strength was defined as a grip strength of less than 28.0 kg in males and less than 18.0 kg in females.

#### 2.4.3. Physical Performance

The 6-meter walk test involves setting aside 2 m at each end of a 10-meter straight line and allowing the subject to complete the test at their individual usual walking speed, recording the time taken for the middle 6 m of the walk. The criteria for low physical performance included that the subject’s result in the 6-meter walk test was less than 1 m/s.

### 2.5. Statistical Analysis

All quantitative data are expressed as mean ± standard deviation, and qualitative data are expressed as frequencies (*n*) and percentages (%). Participants were categorized into two and four groups based on whether they had sarcopenia and their DII score quartiles. The Chi-square test was used to compare participant characteristic data in the sarcopenia subgroup. ANOVA was used to compare food consumption and sarcopenia symptoms between the DII groups. Multifactorial logistic regression models were used to explore the relationship between DII, sarcopenia and its components. Using the lowest quartile as a reference, the calculated odds ratios and 95% confidence intervals are represented as ORs and CI. All data were analyzed using SAS 9.4 and *p* < 0.05 was considered significant.

## 3. Results

### 3.1. Characteristics of the Subjects

This study included a total of 993 subjects, 57% of whom were female, with a mean age of 71.2 ± 5.1 years. The prevalence of sarcopenia diagnosed according to AWGS 2019 criteria was 20.2%, and the characteristics of subjects grouped according to the sarcopenia status are shown in Table 1. The mean Dietary Inflammatory Index score was 0.99 ± 0.1, with a higher score being observed in the sarcopenia group (1.37 ± 1.63) compared to the non-sarcopenia group (0.90 ± 1.75). Significant differences were found between subjects with and without sarcopenia in terms of age group, BMI, central obesity status and physical activity. The prevalence of sarcopenia was higher in older age groups and lower among those with sufficient weekly hours of moderate to high-intensity physical activity (*p* < 0.05). There were significant differences in gender, age group, alcohol consumption, BMI group and physical activity among subjects in different Dietary Inflammatory Index score groups (*p* < 0.05). The results are presented in Table S1 of the Supplementary Materials.

### 3.2. Food Consumption of Four DII Groups

The food consumption of the participants grouped by DII score is shown in Table 2. Participants were categorized into four groups based on DII score quartiles: Q1 (−3.51 to −0.20), Q2 (−0.19 to 1.25), Q3 (1.25 to 2.32) and Q4 (2.32 to 4.63). The study’s findings indicated that significant differences in the consumption of food groups, except for livestock meats, were observed among all DII groups (*p* < 0.05). In the highest quartile of DII scores, the consumption of all food groups except animal oils was significantly lower than in the lowest quartile. In contrast, consumption of animal oils was significantly higher in the highest DII quartile than in the lowest DII quartile (*p* < 0.05).

### 3.3. Sarcopenia Symptomology

Significant differences in handgrip strength, step speed and muscle mass were observed across the different DII groups (*p* < 0.05). Participants in higher DII score groups had a lower grip strength, step speed, upper and lower limb and trunk muscle mass and SMI (*p* < 0.05). These results are shown in Table 3.

### 3.4. Associations Between DII and Sarcopenia

Multifactorial Logistic regression was employed to analyze the relationship between DII and the risk of sarcopenia, with the results presented in Table 4. In the unadjusted model, the Q4 group exhibited a significantly higher risk of sarcopenia compared to the Q1 group (*p-trend* < 0.05), with a statistically significant difference and the DII scores had a significant positive correlation (*p-trend* < 0.05). Model Ⅱ, adjusted for the confounders age, gender, marital status and education, indicated that the risk of muscle wasting in the Q4 group was 1.66 times higher than in the Q1 group (95% CI: 1.04–2.66, *p* < 0.05), with DII scores still significantly and positively correlated with sarcopenia (*p-trend* < 0.05). Model Ⅲ adjusted for alcohol consumption, smoking, physical activity level and chronic diseases in addition to the Model 2 confounders, and the results of Model 3 showed no statistically significant difference in group Q4 compared to group Q1.

### 3.5. Association of DII with Muscle Mass, Grip Strength and Physical Performance

The results of further analyzing the association of DII with muscle mass, strength and physical performance are shown in Table 5. The findings indicate that, in the adjusted logistic regression model, the Q4 group had a significantly higher risk of low grip strength compared to the Q1 group (ORs: 1.65, 95% CI: 1.04–2.61, *p* < 0.05). And DII was significantly and positively correlated with low grip strength (*p-trend* < 0.05). But the correlation between DII and muscle mass or physical function was not statistically significant (*p* > 0.05).

## 4. Discussion

In this study, a cross-sectional survey was conducted on a population of community-dwelling older adults aged 65 and above across four provinces in China. A total of 993 participants were enrolled to assess the prevalence of sarcopenia in this population. The prevalence of sarcopenia in these subjects was 20.2% according to the AWGS 2019 diagnostic criteria. Their food consumption was investigated using the FFQ. The DII was used to assess dietary inflammatory potential and investigate its associations with sarcopenia and its components. The results of the correlation study indicated that while there was no relationship between DII and sarcopenia after model adjustment, higher DII scores were associated with a decrease in muscle strength in the analyses concerning the components of sarcopenia.

The risk of sarcopenia is influenced by several factors, with age being a key determinant in its development. The global prevalence of sarcopenia among older adults remains high and varies considerably depending on the consensus definitions used. A systematic review of the world reported that the prevalence of sarcopenia in people aged 60 and above, according to six different consensus definitions of sarcopenia, ranged from 10% to 27% [30]. According to the data published by the AWGS, the prevalence of sarcopenia in older adults in Asia is between 5.5% and 25.7% [29]. As China has entered a stage of deep aging, the health of the elderly population is of paramount concern. A systematic review of sarcopenia in community-dwelling elderly people in China found an overall prevalence of 17.4% in individuals aged 65 years or older [31]. In this study, the prevalence of sarcopenia among older adults in four provinces of China was found to be 20.2%, which was consistent with the results of previous studies. This study also found that the prevalence was higher in the older age group and underweight group, and lower in those who had sufficient weekly hours of physical activity. An epidemiological study from China reported that the prevalence of sarcopenia increases with age in the elderly [32]. In addition, appropriate physical activity, especially resistance exercise, can promote muscle health and prevent sarcopenia [33].

During the aging process, the body experiences a constant state of low-level inflammation, and previous studies have suggested that this age-related low-level chronic inflammation may play a crucial role in the pathogenesis of sarcopenia [34,35]. The reduction in skeletal muscle mass in inflammatory states is associated with an increase in pro-inflammatory cytokines, which contribute to sarcopenia by enhancing protein hydrolysis and inhibiting protein synthesis in muscle [9]. Pro-inflammatory factors involved in inflammation promote inflammatory cell infiltration through activation of the transcription factor NF-kB leading to muscle attenuation [36]. NLRP3 inflammatory vesicles are now recognized as key regulators of metabolic inflammation and can induce inflammation-induced muscle atrophy through the activation of IL-1β [37]. Furthermore, pro-inflammatory cytokines can suppress muscle protein anabolism by inhibiting the Akt/mTOR signaling pathway [38]. Inflammation impacts both the proteolytic and anabolic metabolism of skeletal muscle, impairing muscle health by increasing degradation and inhibiting synthesis, ultimately leading to muscle atrophy.

Diet is vital in regulating the degree of inflammation within the body [12]. The Dietary Inflammatory Index (DII) is a tool that quantitatively evaluates the overall inflammatory effect of diet and has demonstrated a superior ability to reflect the influence of diet on inflammation levels [39]. In this study, the DII scores of the study population ranged from −3.51 to 4.63. Subjects were divided into four groups based on DII score quartiles. Participants with sarcopenia exhibited notably higher DII scores than those without sarcopenia. Populations with higher DII score had a higher prevalence of sarcopenia, a higher proportion of females, a lower level of education and a lower proportion of habitual alcohol drinkers and as well as a lower proportion of individuals engaging in more than 150 min of moderate-intensity or higher-intensity exercise per week. These findings are consistent with some previous studies [40], which may be due to the fact that people with lower levels of education are more likely to lack knowledge about rational diet, lack rationality and variety in food choices, and that unhealthy lifestyle habits are often associated with poor dietary habits. Additionally, those with higher DII levels consumed more animal oils and less coarse grains, legumes, vegetables and fruits. Previous studies have shown that Western dietary patterns characterized by an inadequate consumption of vegetables and dietary fiber are associated with increased levels of chronic inflammation [41,42]. Similarly, an excessive intake of animal oils may also contribute to increased levels of chronic inflammation [43].

The DII has been extensively utilized to evaluate the impact of dietary inflammation and its relationship to muscle health. Jiwen Geng et al. [23] used data from the US NHANCES study (1999–2006), demonstrated a positive correlation between DII and the risk of sarcopenia. A cross-sectional study of an elderly population in the Shanghai community [26] demonstrated a relationship between inflammatory diet and sarcopenia, but this association was primarily due to a low dietary intake of energy, protein, and anti-inflammatory foods rather than a high intake of pro-inflammatory foods. A meta-analysis also showed significant associations between DII and an increased risk of sarcopenia, low muscle strength, weakness and disability, with a linear dose–response relationship indicating a 14% increase in sarcopenia risk, a 6% increase in low muscle mass risk and a 7% increase in the risk of low muscle strength per one-point increase in the DII score [44]. In our study, after adjusting for confounding factors, including gender, age, education and marital status, we found that the risk of sarcopenia in the group with the highest DII score was 1.66 times higher than that in the group with the lowest score. However, this association was not observed after additional confounding factors such as smoking state, physical activity level and chronic disease status were taken into account. The findings of existing studies suggest that sarcopenia is notably associated with a variety of chronic conditions, the presence of chronic conditions in the elderly may significantly influence muscle status [45]. Adjusting for the prevalence of chronic conditions, these factors may also attenuate a portion of the variance between the Dietary Inflammation Index and sarcopenia, resulting in the disappearance of a significant relationship that would not otherwise exist. Similarly, a cohort study from Hong Kong also showed no association between DII and sarcopenia in 3995 community-dwelling older adults aged 65 and above [27]. Overall, despite the differences in the types of studies, sample sizes and methods and criteria used for the detection of sarcopenia in several existing studies from different populations, and inconsistencies in research findings, the DII may still be a risk factor for the development of sarcopenia. Although some studies have not observed a correlation between DII and sarcopenia, its association with indicators of muscle health should not be disregarded and there is a need to further explore the association between DII and sarcopenia components.

In the present study, we conducted a deeper analysis of the association between DII and sarcopenia components. After adjusting for the key confounding factors, a higher DII score was only associated with an increased risk of decreased muscle strength. Similarly, a study in Iran reported that higher DII levels were associated with lower muscle strength and endurance in Iranian adults [46]. A significant association between higher DII levels and the risk of low muscle strength was also found in a health and nutrition survey in Korea [47]. Furthermore, some research indicates that DII may be linked to other indicators of muscle health besides muscle strength. A cohort study in Hong Kong found the same results as this study. Although no association was found between DII and sarcopenia, consumption of an inflammatory diet promoted diminished muscle strength in elderly in a study of the components of sarcopenia [27]. In addition, UK Biobank research identified a notable association between the DII and sarcopenia, as well as all three of its components [48], and the same conclusion is also supported by Corey Linton et al. [42].

Our study found that higher DII scores were associated with lower muscle strength in an older population. The findings suggest that older adults who adhered more to relatively anti-inflammatory diets had lower rates of low muscle strength compared with those who adhered less to diets with a low dietary inflammatory index. One of the strengths of this study is that it focuses on the current lack of sufficient epidemiological evidence in studies on dietary inflammation index and sarcopenia. This study was carried out with a population aged 65 and above across four regionally representative provinces in China, providing evidence from the Chinese population to explore the relationship between DII and sarcopenia.

This study also recognizes certain limitations. Since the conclusions are drawn from cross-sectional data, a causal relationship between dietary inflammatory potential and sarcopenia could not be established. Future research should include more cohort studies and intervention trials to further investigate the association between dietary inflammation and sarcopenia. In addition, this study is based on a population of community-dwelling older adults in China, and there may be limitations in extrapolating our results to other countries. The diagnosis of muscle mass in this study was measured by the bioresistive method, although it is not the gold-standard method. However, this method has been shown to have a high concordance with the gold-standard diagnostic results and is still widely used in investigations related to sarcopenia, and is therefore still feasible [49]. Finally, this study used an FFQ to investigate the consumption of various foods over the past year, but it is still not possible to avoid recall bias and other unidentified confounding factors of inaccurate food and nutrient intake, which can lead to bias in the calculation of the DII.

## 5. Conclusions

This study evaluated the inflammatory potential of diets based on the DII and explored the association between the DII and sarcopenia and its components. The results indicated that higher levels in the DII were associated with poorer muscle strength. Reducing the inflammatory potential of the diet may be important for preventing the occurrence of sarcopenia and promoting muscle health in the elderly population. These findings also provide new insights for developing dietary intervention strategies aimed at preventing and managing sarcopenia.

## Figures and Tables

**Table 1:** Participants’ characteristics.

	ALL (*n* = 993)	Sarcopenia (*n* = 201)	Non-Sarcopenia (*n* = 792)	χ^2^/*t*	*p*
DII	0.99 ± 0.1	1.37 ± 1.63	0.90 ± 1.75	−3.48	<0.01
Gender				<0.01	0.99
Male	469 (47.2)	95 (20.3)	374 (79.7)		
Female	524 (52.8)	106 (20.2)	418 (79.8)		
Age				73.16	<0.01
<70	448 (45.1)	50 (11.2)	398 (88.8)		
70~79	475 (47.8)	114 (24.0)	361 (76.0)		
80~	70 (7.1)	37 (52.9)	33 (4.2)		
Education				5.79	0.06
Primary school	629 (63.4)	142 (22.6)	487 (77.4)		
Junior Secondary	203 (20.4)	33 (16.3)	170 (83.7)		
Senior Secondary	161 (16.2)	26 (16.2)	135 (83.8)		
Marital Status				4.37	0.22
Married	772 (77.7)	139 (18.0)	633 (82.0)		
Single or divorced	221 (22.3)	62 (28.0)	159 (72.0)		
Smoking				3.07	0.08
No	758 (76.3)	144 (19.0)	614 (81.0)		
Yes	235 (23.7)	57 (24.3)	58 (75.7)		
Drinking				2.47	0.11
No	722 (72.7)	155 (21.5)	567 (78.5)		
Yes	271 (27.3)	46 (17.0)	225 (83.0)		
BMI				135.59	<0.01
Low	57 (5.7)	34 (59.6)	23 (40.4)		
Normal	476 (47.9)	139 (29.2)	337 (70.8)		
Overweight	343 (34.6)	21 (6.1)	322 (93.9)		
Obesity	117 (11.8)	7 (6.0)	110 (94.0)		
Central obesity				87.9	<0.01
No	586 (59.0)	177 (30.2)	409 (69.8)		
Yes	407 (41.0)	24 (5.9)	383 (94.1)		
NCDs					
Diabetes	199 (20.0)	34 (17.1)	165 (82.9)	1.54	0.22
Hypertension	482 (48.5)	96 (19.9)	386 (80.1)	0.06	0.80
Dyslipidemia	505 (50.9)	87 (17.2)	418 (82.8)	5.78	0.02
Exercise activity (min/week)				7.23	<0.01
Less than 150	844 (85.0)	183 (21.7)	661 (78.3)		
More than 150	149 (15.0)	18 (12.1)	131 (87.9)		
Sleeping time				0.01	0.93
<7 h	299 (30.1)	60 (20.1)	239 (79.9)		
≥7 h	694 (69.9)	141 (20.3)	553 (79.7)		
Sedentary time				1.41	0.24
<5 h	427 (43.0)	79 (18.5)	348 (81.5)		
≥5 h	566 (57.0)	122 (21.6)	444 (78.4)		

**Table 2:** Food consumption of four DII groups.

Food Group	Q1	Q2	Q3	Q4	*F*	*p*
Rice	136.8 ± 82.8	134.2 ± 87.2	132.5 ± 82.4	96.3 ± 76.0	13.53	<0.01
Wheat	78.7 ± 74.6	82.0 ± 82.4	65.3 ± 74.6	58.7 ± 65.6	5.42	<0.01
Coarse cereals	40.1 ± 57.9	23.7 ± 38.4	19.9 ± 35.1	16.7 ± 34.5	14.77	<0.01
Tubers	13.5 ± 25.4	10.1 ± 26.6	3.8 ± 13.4	1.3 ± 3.7	20.22	<0.01
Soybean and products	22.0 ± 37.6	14.5 ± 15.6	10.2 ± 10.1	6.3 ± 7.7	24.70	<0.01
Legumes	39.8 ± 60.4	40.1 ± 65.1	32.1 ± 55.9	26.1 ± 33.7	3.68	0.01
Vegetable	765.1 ± 427.5	501.8 ± 235.6	364.8 ± 192.0	210.1 ± 146.2	185.60	<0.01
Fruits	163.8 ± 154.4	122.2 ± 107.6	89.1 ± 90.3	59.5 ± 63.4	41.91	<0.01
Mushrooms and fungi	31.7 ± 57.9	14.1 ± 22.4	9.3 ± 12.4	5.4 ± 7.9	32.72	<0.01
Pork	56.9 ± 62.1	59.2 ± 73.6	38.9 ± 41.7	39.6 ± 45.9	8.95	<0.01
Livestock meats	12.4 ± 29.6	15.3 ± 39.5	11.8 ± 25.3	10.1 ± 20.4	1.33	0.26
Poultry	25.2 ± 41.1	19.7 ± 42.3	13.2 ± 30.2	12.8 ± 25.0	6.91	<0.01
Animal viscera	1.8 ± 5.8	1.4 ± 3.9	0.9 ± 3.4	0.5 ± 1.6	4.94	<0.01
Fish and seafood	73.8 ± 82.4	52.4 ± 85.9	26.6 ± 30.8	19.1 ± 27.2	39.14	<0.01
Egg	51.5 ± 50.1	47.0 ± 45.8	41.9 ± 40.4	40.3 ± 29.8	3.62	0.01
Milk	123.9 ± 186.8	99.4 ± 140.2	75.9 ± 128.1	49.8 ± 83.1	12.84	<0.01
Soft drinks	87.3 ± 274.8	7.2 ± 30.7	6.5 ± 22.5	8.0 ± 41.2	20.25	<0.01
Snacks	39.9 ± 61.0	29.1 ± 42.8	23.5 ± 32.2	15.4 ± 25.7	14.41	<0.01
Alcoholic beverages	47.8 ± 176.2	55.1 ± 169.8	37.6 ± 153.5	7.3 ± 47.5	5.12	<0.01
Animal oil	1.9 ± 6.1	6.1 ± 12.0	7.0 ± 14.0	10.9 ± 19.2	17.94	<0.01
Vegetable oil	31.4 ± 26.4	28.2 ± 21.8	29.6 ± 21.4	18.2 ± 16.5	18.47	<0.01

**Table 3:** Sarcopenia symptomology of DII groups.

	Q1	Q2	Q3	Q4	*F*	*p*
Handgrip strength	28.93 ± 8.75	26.33 ± 7.86	25.90 ± 9.09	23.34 ± 8.60	17.64	<0.01
Step speed	1.07 ± 0.47	1.03 ± 0.23	0.98 ± 0.23	0.92 ± 0.25	11.09	<0.01
Upper limb muscle mass	4.42 ± 1.12	4.36 ± 1.15	4.22 ± 1.12	4.06 ± 1.09	5.11	<0.01
Trunk muscle mass	19.26 ± 3.47	19.02 ± 3.57	18.53 ± 3.49	17.99 ± 3.40	6.51	<0.01
Lower limb muscle mass	12.91 ± 2.85	12.65 ± 3.02	12.25 ± 2.89	11.86 ± 2.72	6.37	<0.01
SMI	6.66 ± 0.95	6.61 ± 1.01	6.54 ± 1.00	6.37 ± 0.99	4.03	0.01

**Table 4:** Associations between DII and sarcopenia.

	Model Ⅰ		Model Ⅱ		Model Ⅲ	
DII	ORs (95% CI)	*p*	ORs (95% CI)	*p*	ORs (95% CI)	*p*
		<0.01		0.02		0.10
Q1	1.00		1.00		1.00	
Q2	0.97 (0.60, 1.56)	0.89	1.02 (0.62, 1.69)	0.93	1.06 (0.62, 1.82)	0.83
Q3	1.28 (0.81, 2.01)	0.30	1.23 (0.76, 2.01)	0.40	1.25 (0.73, 2.12)	0.42
Q4	1.99 (1.29, 3.07)	<0.01	1.66 (1.04, 2.66)	0.03	1.49 (0.89, 2.50)	0.13

Model Ⅰ: unadjusted model. Model Ⅱ: adjusted for age, gender, marital status, and education. Model Ⅲ: additional adjustment for BMI, exercise activity, sleeping time, sedentary time and smoke status based on Model 2.

**Table 5:** Associations between DII and sarcopenia symptomology.

	Muscle Mass		Grip Strength		Physical Performance	
DII	ORs (95% CI)	*p*	ORs (95% CI)	*p*	ORs (95% CI)	*p*
		0.57		0.04		0.15
Q1	1.00		1.00		1.00	
Q2	1.25 (0.79, 1.99)	0.34	1.39 (0.87, 2.22)	0.17	0.69 (0.47, 1.03)	0.07
Q3	1.04 (0.65, 1.67)	0.87	1.52 (0.96, 2.41)	0.07	0.94 (0.64, 1.38)	0.76
Q4	1.23 (0.76, 2.00)	0.39	1.65 (1.04, 2.61)	0.03	1.20 (0.81, 1.78)	0.37

This model was adjusted for age, gender, marital status, education, BMI, exercise activity, sleeping time, sedentary time and smoking status.

## Data Availability

The datasets presented in this article are not readily available. Requests to access the datasets should be directed to www.chinanutri.cn.

## Supplementary Materials

The following supporting information can be downloaded at: https://www.mdpi.com/article/10.3390/nu17030478/s1, Table S1: Participants characteristics of DII groups; Table S2: Association of DII with muscle mass, grip strength, and physical performance.

## Abbreviations

DII: Dietary Inflammatory Index; FFQ: food frequency questionnaire; BMI: body mass index; NCDs: non-communicable chronic diseases; AWGS2019: Asian Working Group for Sarcopenia in 2019; ASM: appendicular skeletal muscle mass; SMI: skeletal muscle index; ORs: odds ratios; CI: confidence interval.
