# Supplementary material for: The Dietary Inflammatory Index and Sarcopenia in Older Adults in Four Chinese Provinces: A Cross-Sectional Study
Source: Nutrients. 2025 Jan 28;17(3):478. doi: 10.3390/nu17030478 (PMC11820900; doi:10.3390/nu17030478)
Supplement: Supplementary file 1 [file nutrients-17-00478-s001.zip › nutrients-3440922-supplementary.pdf]

Supplementary Material

Table S1 Participants characteristics of dietary inflammatory index groups

	ALL (n=993)	Q1 (n=248)	Q2 (n=249)	Q3 (n=248)	Q4 (n=248)	χ^2	*P*
Sarcopenia						14.60	<0.01
Yes	201(20.2)	41(16.5)	40(16.1)	50(20.2)	70(28.2)		
No	792(79.8)	207(83.5)	209(83.9)	198(79.8)	178(71.8)		
Gender						13.38	<0.01
Male	469(47.2)	141(56.9)	115(46.2)	110(44.3)	103(41.5)		
Female	524(52.8)	107(42.1)	134(53.8)	138(55.7)	145(58.5)		
Age						26.66	<0.01
<70	448(45.1)	122(49.2)	132(53.0)	112(45.2)	82(33.1)		
70~79	475(47.8)	108(43.5)	106(42.6)	122(49.2)	139(56.0)		
80~	70(7.1)	18(7.3)	11(4.4)	14(5.6)	27(10.9)		
Education						88.66	<0.01
Primary school	629(63.4)	103(41.5)	160(64.2)	176(71.0)	190(76.6)		
Junior secondary	203(20.4)	67(27.0)	51(20.5)	49(19.7)	36(14.5)		
Senior secondary	161(16.2)	78(31.5)	38(15.3)	23(9.3)	22(8.9)		
Marital Status						4.37	0.22
Married	772(77.7)	200(80.6)	200(80.3)	185(74.6)	187(75.4)		
Single or divorced	221(22.3)	48(19.4)	49(19.7)	63(25.4)	61(24.6)		
Smoking						0.96	0.81
No	758(76.3)	193(77.8)	191(76.7)	190(76.6)	184(74.2)		
Yes	235(23.7)	55(22.2)	58(23.3)	58(23.4)	64(25.8)		
Drinking						69.9	<0.01
No	722(72.7)	139(56.1)	173(69.5)	190(76.6)	220(88.7)		
Yes	271(27.3)	109(43.9)	76(30.5)	58(23.4)	28(11.3)		
BMI						22.45	0.01
Low	57(5.7)	13(5.2)	8(3.2)	9(3.6)	27(10.9)		
Normal	476(47.9)	124(50.0)	120(48.2)	123(49.6)	109(43.9)		
Overweight	343(34.6)	88(35.5)	84(33.7)	91(36.7)	80(32.3)		
Obesity	117(11.8)	23(9.3)	37(14.9)	25(10.1)	32(12.9)		
Central obesity						2.18	0.54
No	586(59.0)	143(57.7)	142(57.0)	145(58.5)	156(62.9)		
Yes	407(41.0)	105(42.3)	107(43.0)	103(41.5)	92(37.1)		
NCDs							
Diabetes	199(20.0)	54(21.8)	47(18.9)	42(16.9)	56(22.6)	3.17	0.37
Hypertension	482(48.5)	120(48.4)	112(45.0)	129(52.0)	121(48.8)	0.83	0.66
Dyslipidemia	505(50.9)	134(54.0)	130(52.2)	116(46.8)	125(50.4)	2.86	0.41
Exercise activity						39.14	<0.01
<150 min/week	844(85.0)	187(75.4)	203(81.5)	221(89.1)	233(93.9)		
≥150 min/week	149(15.0)	61(24.6)	46(18.5)	27(10.9)	15(6.1)		

Sedentary time						4.30	0.23
<5h	427(43.0)	95(38.3)	104(41.8)	116(46.8)	112(45.2)		
⩾5h	566(57.0)	153(61.7)	145(58.2)	132(53.2)	136(54.8)		
Sleeping time						6.04	0.11
<7h	299(30.1)	90(36.3)	71(28.5)	69(27.8)	69(27.8)		
⩾7h	694(69.9)	158(63.7)	178(71.5)	179(72.2)	179(72.2)		

Categorical data are shown as n (%); Abbreviations: BMI—Body-mass index; NCDs—Non-communicable chronic diseases.

Table S2 Association of dietary inflammatory index with muscle mass, grip strength, and physical performance

	Model 1		Model 2		Model 3	
DII	OR (95%CI)	*P*	OR (95%CI)	*P*	OR (95%CI)	*P*
			Muscle mass			
Q1	Ref		Ref		Ref	
Q2	0.93(0.64,1.34)	0.68	1.10(0.74,1.61)	0.64	1.25(0.79,1.99)	0.34
Q3	0.88(0.61,1.28)	0.51	1.00(0.68,1.49)	0.99	1.04(0.65,1.67)	0.87
Q4	1.29(0.90,1.85)	0.17	1.33(0.90,1.98)	0.15	1.23(0.76,2.00)	0.39
			Grip strength			
Q1	Ref		Ref		Ref	
Q2	1.56(1.02,2.40)	0.04	1.44(0.91,2.28)	0.12	1.39(0.87,2.22)	0.17
Q3	1.99(1.31,3.04)	**<0.01**	1.62(1.03,2.55)	**0.04**	1.52(0.96,2.41)	0.07
Q4	2.71(1.79,4.10)	**<0.01**	1.91(1.22,2.99)	**<0.01**	1.65(1.04,2.61)	**0.03**
			Physical performance			
Q1	Ref		Ref		Ref	
Q2	0.86(0.60,1.25)	0.43	0.76(0.52,1.11)	0.16	0.69(0.47,1.03)	0.07
Q3	1.21(0.84,1.73)	0.31	0.98(0.67,1.44)	0.93	0.94(0.64,1.38)	0.76
Q4	1.73(1.21,2.47)	**<0.01**	1.31(0.89,1.91)	0.17	1.20(0.81,1.78)	0.37

Model 1: Crude model. Model 2: adjusted by age, gender, and region. Model 3: adjusted by age, gender, region, body mass index (BMI), exercise activity, sleeping time, sedentary time, and smoke status. DII score ranged from −3.51 to 4.63; Q1 (−3.51~−0.20), Q2 (−0.19~1.25), Q3 (1.25~2.32), Q4 (2.32~4.63). BMI—Body mass index.DII—Dietary inflammatory index.
